# Association between body mass index and vitiligo distribution: An observational cohort study

**DOI:** 10.1016/j.jdin.2024.03.029

**Published:** 2024-05-04

**Authors:** Ross O’Hagan, Samir Kamat, Shira Wieder, Marcel Perl, Jonathan I. Silverberg, Nanette B. Silverberg

**Affiliations:** a Department of Dermatology, Icahn School of Medicine at Mount Sinai, New York, New York; b Department of Dermatology, George Washington University School of Medicine and Health Sciences, Washington, District of Columbia

**Keywords:** BMI, BSA, metabolic syndrome, overweight, vitiligo

*To the Editor:* Vitiligo is an inflammatory skin condition affecting 0.5% to 2%^1^ of the population. A recent meta-analysis of 28,325 patients with vitiligo identified association with diabetes mellitus and obesity.^2^ The direct effect of weight on vitiligo severity has yet to be elucidated. We hypothesized that with greater body mass index (BMI) there is greater skin friction, which could result in Koebner phenomenon on the central portion of the body.

Patients self-reported their sex, age, race, vitiligo regions of distribution, height, and weight. Only respondents providing both height and weight as well as proper units were included for analysis. BMI was calculated using the formula: weight (lb)/(height [in])^2^ × 703 (kg/m^2^). BMI classifications included underweight for patients with a BMI <18, normal for patients ≥18 but <25, overweight for >25 and <30, and obese for BMI ≥30. Analysis was performed with R (version 1.4).

The cohort (*n* = 1685) had a median age of 42 years, was predominantly female (72%), and White (64%), of which there were underweight (*n* = 51), normal weight (*n* = 820), overweight (*n* = 344), and obese (*n* = 344) individuals. As weight classification increased, vitiligo presentation (Table I) was more likely on the chest (*P* =.020), stomach (*P* <.001), axillae (*P* =.005), arms (*P* =.023), elbows (*P* =.048), wrists (*P* =.040), hands (*P* =.004), fingers (*P* <.001), genitals (*P* =.028), buttocks (*P* =.0015), ankles (*P* =.012), feet (*P* =.004), and toes (*P* =.011). Vitiligo presentation on the eyelids, lips, mouth, back, hips, legs, and knees did not have a relationship with one’s BMI classification.

**Table I:** Characteristics of cohort stratified by weight classification

Variable	*N*	Underweight, *N* = 51∗	Normal, *N* = 820∗	Overweight, *N* = 470∗	Obese, *N* = 344∗	*P* Value
Eyelids	1420	20 (45)	354 (52)	201 (51)	177 (58)	.2
Lips	1387	14 (33)	258 (39)	163 (43)	121 (41)	.4
Mouth	1250	3 (7.0)	51 (8.5)	38 (11)	31 (12)	.4
Chest	1429	27 (59)	407 (59)	227 (58)	207 (68)	.020
Stomach	1385	15 (36)	316 (48)	187 (49)	195 (65)	<.001
Back	1375	17 (40)	391 (58)	216 (58)	154 (54)	.081
Axillae	1462	26 (58)	477 (68)	303 (74)	238 (77)	.005
Arms	1461	23 (53)	486 (69)	298 (72)	227 (75)	.023
Elbows	1467	28 (65)	496 (70)	285 (71)	242 (78)	.048
Wrists	1469	27 (63)	519 (74)	320 (78)	251 (80)	.040
Hands	1532	30 (70)	584 (79)	368 (85)	281 (87)	.004
Fingers	1514	27 (64)	564 (77)	361 (85)	273 (86)	<.001
Hips	1354	23 (53)	371 (56)	191 (52)	177 (61)	.2
Genitals	1473	34 (74)	502 (71)	312 (77)	245 (79)	.028
Buttocks	1316	22 (50)	264 (42)	160 (45)	150 (53)	.015
Legs	1468	35 (76)	499 (71)	306 (74)	236 (78)	.11
Knees	1405	24 (52)	424 (63)	248 (64)	202 (68)	.2
Ankles	1408	22 (50)	424 (63)	262 (67)	212 (71)	.012
Feet	1467	23 (53)	491 (70)	296 (72)	240 (78)	.004
Toes	1372	20 (44)	341 (52)	213 (57)	183 (62)	.011

∗ *n* (%).

We found a significant stepwise relationship with the increasing presence of stomach and underarm lesions linked to increasing BMI quartile (Fig 1). For the first quartile, a 46% occurrence of vitiliginous lesions on the stomach was noted, but for the fourth quartile there was a 63% occurrence (*P* <.001). Similarly, for the underarms, the first quartile had 63% occurrence, and the fourth quartile had a 76% occurrence of vitiliginous lesions (*P* <.001).

**Fig 1:**
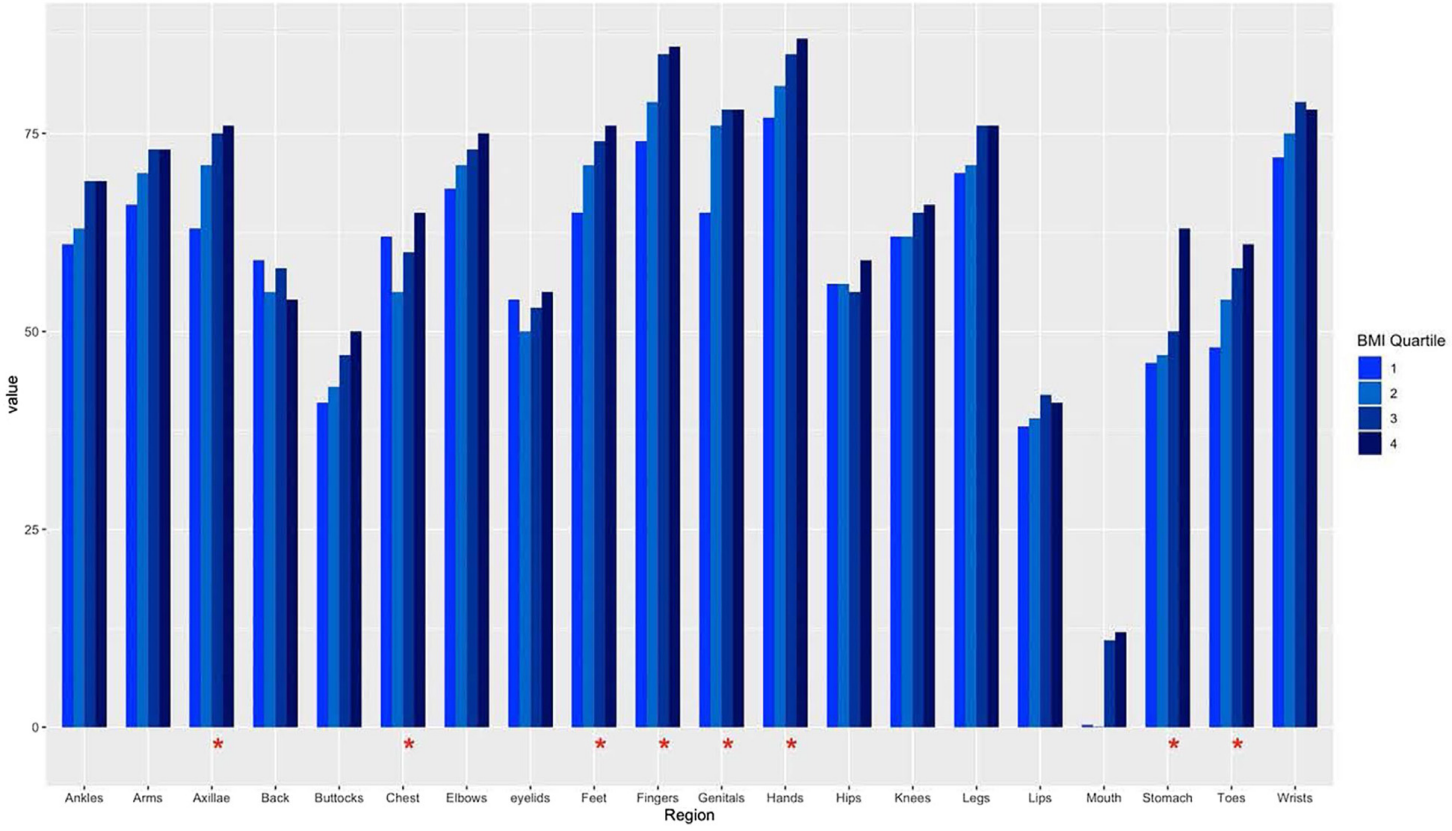
Quartile trends in localization and body mass index quartiles.

The effect of weight on vitiligo extent appears to be a promotional effect. Two recent studies examining patients with type 2 diabetic noted vitiligo in 12% vs 6% of nondiabetics in India, and 4.9% in diabetics^3^ vs 1.8% of nondiabetic patients in Iran.^4^ A picture is created of vitiligo aggravation by higher BMI in sites of dependence and friction, including folds, waistline, feet, and toes.

There are benefits and limitations identified within the survey process. Inherent in all survey processes is the possibility that patients may not provide accurate information. Specifically, it has been demonstrated that patient self-assessment of vitiligo is often not in agreement with physician estimates.^5^ To reduce this risk, we focused on self-identification by disease localization.

The relationship between vitiligo, the Koebner phenomenon, and inflammation in the metabolic syndrome requires prospective study. Our findings support prior data showing an association of vitiligo extent and metabolic syndrome. We demonstrated that increased BMI quartile confers increased risk of central disease (axillae, abdomen, chest, and arms). It remains to be seen if weight control can help control disease spread.

## Conflicts of interest

Dr N. B. Silverberg has been an adviser or received honoraria from Amryt, Incyte, Lilly, Regeneron/Sanofi, and Verrica Pharmaceuticals. Dr J. I. Silverberg has received honoraria as a consultant and/or advisory board member for AbbVie, AOBiome, Arcutis, Alamar, Amgen, Arena, Arcutis, Asana, Aslan, BioMX, Biosion, Bodewell, Boehringer Ingelheim, Cara, Castle Biosciences, Celgene, Connect Biopharma, Dermavant, Dermira, DermTech, Eli Lilly, Galderma, GlaxoSmithKline, Incyte, Kiniksa, Leo Pharma, Menlo, Novartis, Optum, Pfizer, RAPT, Regeneron, Sanofi-Genzyme, Shaperon, and Union, speaker for AbbVie, Eli Lilly, Leo Pharma, Pfizer, Regeneron, and Sanofi-Genzyme, and institution received grants from Galderma and Pfizer. The other authors have no conflicts of interest to declare.
